# Two-stage laparoscopic transversus abdominis plane block as an equivalent alternative to thoracic epidural anaesthesia in bowel resection—an explorative cohort study

**DOI:** 10.1007/s00384-023-04592-6

**Published:** 2024-01-11

**Authors:** M. Kaufmann, V. Orth, T.-J. Dorwarth, J. Benrath, B. Gerber, D. Ghezel-Ahmadi, C. Reißfelder, F. Herrle

**Affiliations:** 1 Department of Surgery, Medical Faculty Mannheim, University Medical Centre Mannheim, Heidelberg University, Mannheim, Germany; 2 Department of Anesthesiology, Medical Faculty Mannheim, University Medical Centre Mannheim, Heidelberg University, Mannheim, Germany

**Keywords:** Two-stage laparoscopic transversus abdominis plane block, TAP, L-TAP, Thoracic epidural anaesthesia, Bowel resection

## Abstract

**Purpose:**

We evaluated the effect of the two-stage laparoscopic transversus abdominis plane block (TS-L-TAPB) in comparison to thoracic epidural anaesthesia (TEA) and a one-stage L-TAPB (OS-L-TAPB) in patients who underwent elective laparoscopic bowel resection.

**Methods:**

We compared a TS-L-TAPB (266 mg bupivacaine), which was performed bilaterally at the beginning and end of surgery, with two retrospective cohorts. These were patients who had undergone a TEA (ropivacaine/sufentanil) or an OS-L-TAPB (200 mg ropivacaine) at the beginning of surgery. Oral and i.v. opiate requirements were documented over the first 3 postoperative days (POD).

**Results:**

Patients were divided into three groups TEA (*n* = 23), OS-L-TAPB (*n* = 75), and TS-L-TAPB (*n* = 49). By the evening of the third POD, patients with a TEA had a higher cumulative opiate requirement with a median of 45.625 mg [0; 202.5] than patients in the OS-L-TAPB group at 10 mg [0; 245.625] and the TS-L-TAPB group at 5.625 mg [0; 215.625] (*p* = 0.1438). One hour after arrival in the recovery room, significantly more patients in the TEA group (100%) did not need oral and i.v. opioids than in the TS-L-TAPB (78%) and OS-L-TAPB groups (68%) (*p* = 0.0067).This was without clinical relevance however as the median in all groups was 0 mg. On the third POD, patients in the TEA group had a significantly higher median oral and i.v. opioid dose at 40 mg [0; 80] than the TS-L-TAPB and OS-L-TAPB groups, both at 0 mg [0; 80] (*p* = 0.0009).

**Conclusion:**

The TS-L-TAP showed statistically significant and clinically meaningful benefits over TEA and OS-L-TAP in reducing postoperative opiate requirements.

## Introduction

The ERAS concept has increasingly found its way into clinical practice over the last decade. Minimally invasive colorectal surgery leads to a shortening of the patient’s functional recovery time and length of hospital stay (LoS) [1, 2]. Particularly, multimodal analgesia focusing on opioid-sparing concepts is one of the cornerstones of successful perioperative management of colorectal patients [3, 4]. Opioids, with their known side effects such as respiratory depression, postoperative nausea and vomiting (PONV), and especially delayed return of gastrointestinal function, lead to slower postoperative recovery in abdominal surgery [3]. One of the recommended interventions in the ERAS guidelines to spare opioids while optimising pain control is the additional use of the transversus abdominis plane block (TAPB). This abdominal wall block has been shown to lead to a significant reduction of required opioids, time to first mobilisation, time to return of gastrointestinal function and LoS [5]. Therefore, TAPB is strongly recommended for postoperative pain relief in the current ERAS guidelines for colorectal surgery [6].

The TAPB was developed in 2001 by Rafi et al. as a landmark-based technique for the preoperative application of local anaesthetics in the Triangle of Petit [7]. The aim of the technique is to place a long-acting anaesthetic depot in the layer between the transversus abdominis muscle and the obliquus internus abdominis muscle to block afferent abdominal wall somatic pain from dermatomes T6 to L1 [8]. Two variations of this technique, the ultrasound-guided TAP block (US-TAPB) and the laparoscopically guided intraoperative TAP block (L-TAPB) by the surgeon have been developed [9, 10].

In the current literature, it was not possible to identify a ‘valid best practice’ technique for the TAPB. Notably, one meta-analysis indicated a reduction in opioid requirements and pain scores within the first 24 h with the L-TAPB compared to the US-TAPB [11].

The optimal timing of TAPB is controversial [12]. The direct comparison of US-TAPB showed superior results for either a preoperative [13] or postoperative [14] application of the US-TAPB. There were also studies that did not show any difference between the two options [15, 16]. In a meta-analysis, Hamid et al. described the advantages and disadvantages of a preoperative vs. postoperative application. Preoperative TAPB was associated with better early pain control. In contrast, only postoperative TAPB was able to ensure a significant reduction of required opioids 24 h postoperatively and in the total length of hospital stay [12]. Furthermore, postoperative TAPB offers the possibility to adapt the injections to additional trocars or incisions in case of conversion [15].

Therefore, our study rationale was to combine the advantages of both options by performing a two-stage-intraoperative L-TAPB. Our study aimed to explore the effect of a two-stage L-TAPB in comparison to the TEA and a one-stage L-TAPB. Our hypothesis was that using a two-stage L-TAPB could reduce the need for postoperative oral and intravenous (i.v.) opioid consumption and the LoS.

## Methods

Between April 2021 and April 2022, all consecutive adult patients who underwent elective laparoscopic bowel resection in our ERAS-certified pathway were prospectively screened for the study. Exclusion criteria were GFR < 30 ml/min due to the renal elimination of bupivacaine, ASA score > 3, an existing language barrier, non-compliance, use of morphine and cannabis or psychological distress. For this purpose, the BDI-V form for depression (threshold > 35 points) and the DASS test (thresholds depression ≥ 10, anxiety ≥ 6, stress ≥ 10) were performed preoperatively. Patients were excluded from the analysis if an unplanned TEA was performed on short notice if an intraoperative conversion from laparoscopic to open surgery was required or if the L-TAPB was not performed correctly according to the specified clinical standard (SOP). Correct performance of two-stage L-TAPB was monitored intraoperatively. This two-stage L-TAPB SOP introduced in April 2021 replaced a previous TAPB SOP in which a one-stage L-TAPB was used only at the beginning of laparoscopic surgery.

For the retrospective cohort, all adult patients who underwent elective laparoscopic surgery with bowel resection between September 2019 and September 2020 were evaluated. The exclusion criteria were like the criteria in the prospective cohort which were listed above. Because of the retrospective review, psychological distress was assessed using only the current list of comorbidities and not according to the tests used in the prospective cohort. From this cohort two subgroups were created, one that received a TEA and one that received a one-stage L-TAPB with ropivacaine (total dose 200 mg) according to the in-house standard at that time were evaluated.

The primary endpoint of the study was morphine milligram equivalent (MME) requirement within the first 3 postoperative days (POD0-POD3). We collected the data 60 min after arrival in the recovery room and on the first 3 postoperative days at 8 am and 8 pm. For the retrospective cohort, it was assumed that morning medication was regularly taken at 8 am, based on experience from the prospectively collected data.

A clinically significant reduction in MME was defined as 30% but at least 10 mg as mentioned in other studies [17, 18]. Secondary endpoints were the LoS and the need for administration of metamizole and paracetamol 60 min after arrival in the recovery room. In addition, the indication, duration and type of surgery and whether a stoma was placed were recorded.

In the prospective cohort, patients received a bilateral L-TAPB twice during the procedure under laparoscopic visual control. Visual control was performed to determine the correct layer by forming a slow and distinct bulge between the muscle layers during the application of the L-TAPB. A 21 G needle and 266 mg of bupivacaine (60 ml) diluted 2:1 with 0.9% sodium chloride (30 ml) were used. The L-TAPB was applied after the creation of the pneumoperitoneum at the beginning of surgery and just before draining the gas and removing all trocars before fascial closure. L-TAPB was performed with an injection of 7 ml at three sites per side in the anterior axillary line 2 cm above the spina iliaca, 2 cm above the first injection and 2 cm below the arcus costalis in the medioclavicular line. The execution was considered correct if the L-TAPB was performed as described at the correct sites under visual control.

In the retrospective group, the L-TAPB was also performed bilaterally under laparoscopic control at the same positions as described. However, the retrospective one-stage L-TAPB was only performed during the initial phase of surgery after creation of the pneumoperitoneum. Instead of bupivacaine, 200 mg of ropivacaine (20 ml) was diluted 1:1 with 20 ml sodium chloride 0.9%. Seven milliliters of this solution was applied per injection.

All groups received a standardised anaesthetic regimen according to the house standard for colorectal surgery. The regimen consisted of sufentanil 0.3–0.5 µg/kg body weight and thiopental 3–5 mg/kg body weight (alternatively propofol 2 mg/kg body weight). In addition, patients received S-ketamine 0.5 mg/kg and 2 g metamizole as a short infusion 15 min before skin suture.

The TEA was performed at the level of the intervertebral space 10/11 with an 18 G Tuohy needle under local anaesthesia with 5 ml scandicaine 1%. After that, a test dose of 2 ml of hyperbaric bupivacaine 0.5% was applied. Intraoperatively, TEA was then injected with 4 doses of 3 ml ropivacaine 0.375% each. A pump containing 10 ml ropivacaine 1%, 7.5 ml sufentanil (5 µg/ml) and 32.5 ml sodium chloride 0.9% was then connected. The standard flow rate was initially 6 ml/h but could be varied as needed in the recovery room and on the ward.

The patients received 1000 mg metamizole or 1000 mg paracetamol in the recovery room. An additional dose of 5 mg oral oxycodone or 7.5 mg intravenous piritramide was administered as needed, depending on the consciousness of the patients.

On the ward, the standardised pain regimen consisted of 1000 mg metamizole four times a day. If needed, patients additionally received 1000 mg paracetamol, 7.5 mg piritramide, 5 mg oxycodone or 10 mg oxycodone. The predefined aim of postoperative pain control was to maintain patients at a subjective pain maximum of NRS-score ≤ 3 for pain at rest. The NRS score was routinely recorded by ward nurses three times per day and whenever the patient asked for additional pain medication.

SAS software version 9.4 (SAS Institute, Cary, NC) was used for all statistical analyses. Normally distributed variables were compared using ANOVA analysis and the Kruskal–Wallis test for non-normally distributed characteristics. If possible, the chi-square test was used to compare dichotomous variables, if not, the Fisher test was used. A two-sided significance level of 0.05 level was used for all exploratory analyses. If not otherwise indicated, numbers are given as median with minimal and maximal range.

The study was prospectively registered in the German Clinical Trials Registry (DRKS no. DRKS00024839) and approved by the Ethics Committee II of the University of Heidelberg (Nr.2021–503).

## Results

In the prospective cohort, a total of 133 consecutive patients were screened. Of these, 89 patients fulfilled the inclusion criteria, and 80 patients could be included in the study. Of these 80 patients, 7 patients underwent TEA, 10 surgeries were converted and in 13 cases, the L-TAPB was not performed correctly. One patient proved to be a chronic pain patient who received cannabis therapy at least postoperatively. Thus, a total of 49 patients with two-stage L-TAPB were included in the analysis (group TS-L-TAPB).

Within the retrospective cohort, 204 consecutive patients were screened and 107 patients fulfilled the inclusion criteria. Of these 107 patients, 26 patients received TEA and 81 patients one-stage L-TAPB with ropivacaine. After the exclusion of 9 converted patients, 23 patients with a TEA (TEA group) and 75 patients with one-stage L-TAPB (group OS-L-TAPB) could be evaluated. A total of 147 minimally invasive operated patients were included in the study.

The baseline characteristics of the three study cohorts are shown in Table 1. Performed surgeries and underlying diagnostic groups are listed in Table 2. The study cohorts were comparable in terms of gender and ASA score. The groups were not comparable due to age differences (*p* = 0.0294) (Table 1).

**Table 1:** Patient characteristics

	TEA (*n* = 23)	OS-L-TAPB (*n* = 75)	TS-L-TAPB (*n* = 49)	*p* value
Age	56.17 (± 18.7)	65.76 (± 14.15)	63.22 (± 14.27)	**0.029**
ASA score 1	1 (4%)	11 (15%)	4 (8%)	0.62
ASA score 2	18 (78%)	48 (64%)	36 (74%)	
ASA score 3	4 (18%)	16 (21%)	9 (18%)	
Male	14 (61%)	43 (57%)	25 (51%)	0.68
Female	9 (39%)	32 (43%)	24 (49%)

**Table 2:** Surgery characteristics

	TEA (*n* = 23)	OS-L-TAPB (*n* = 75)	TS-L-TAPB (*n* = 49)	*p* value
Duration of surgery	355.96 (± 148.35)	238.65 (± 106.04)	245.45 (± 114.64)	** < 0.001**
Colorectal cancer	17 (74%)	54 (72%)	37 (76%)	0.91
IBD	6 (26%)	3 (4%)	4 (8%)	**0.010**
Other indication	0 (0%)	18 (24%)	8 (16)	**0.029**
Right hemicolectomy	1 (4%)	25 (33%)	18 (38%)	**0.013**
Left hemicolectomy	0 (0%)	4 (5%)	2 (4%)	0.86
Colectomy	3 (13%)	8 (11%)	5 (10%)	0.93
Rectal resection	11 (48%)	17 (23%)	12 (24%)	0.05
Other procedures	8 (35%)	21 (28%)	12 (24%)	0.66
Stoma creation	18 (78%)	9 (12%)	8 (16%)	** < 0.001**

Significantly, more rectal surgeries were performed in the TEA group, leading to differences in the type of surgery (e.g. more deep rectal resections and fewer right hemicolectomies), duration or indication of surgery (more ulcerative colitis) and frequency of stoma creation between the groups (Table 2).

All three groups had a median MME of 0 mg in the first hour after arrival in the recovery room with the TEA group [0; 0] being significantly superior to the TS-L-TAB [0; 20] and OS-L-TAPB [0; 40] groups (*p* = 0.0055). Since the MME of all groups was 0 mg, this was not of clinical relevance.

**Fig. 1:**
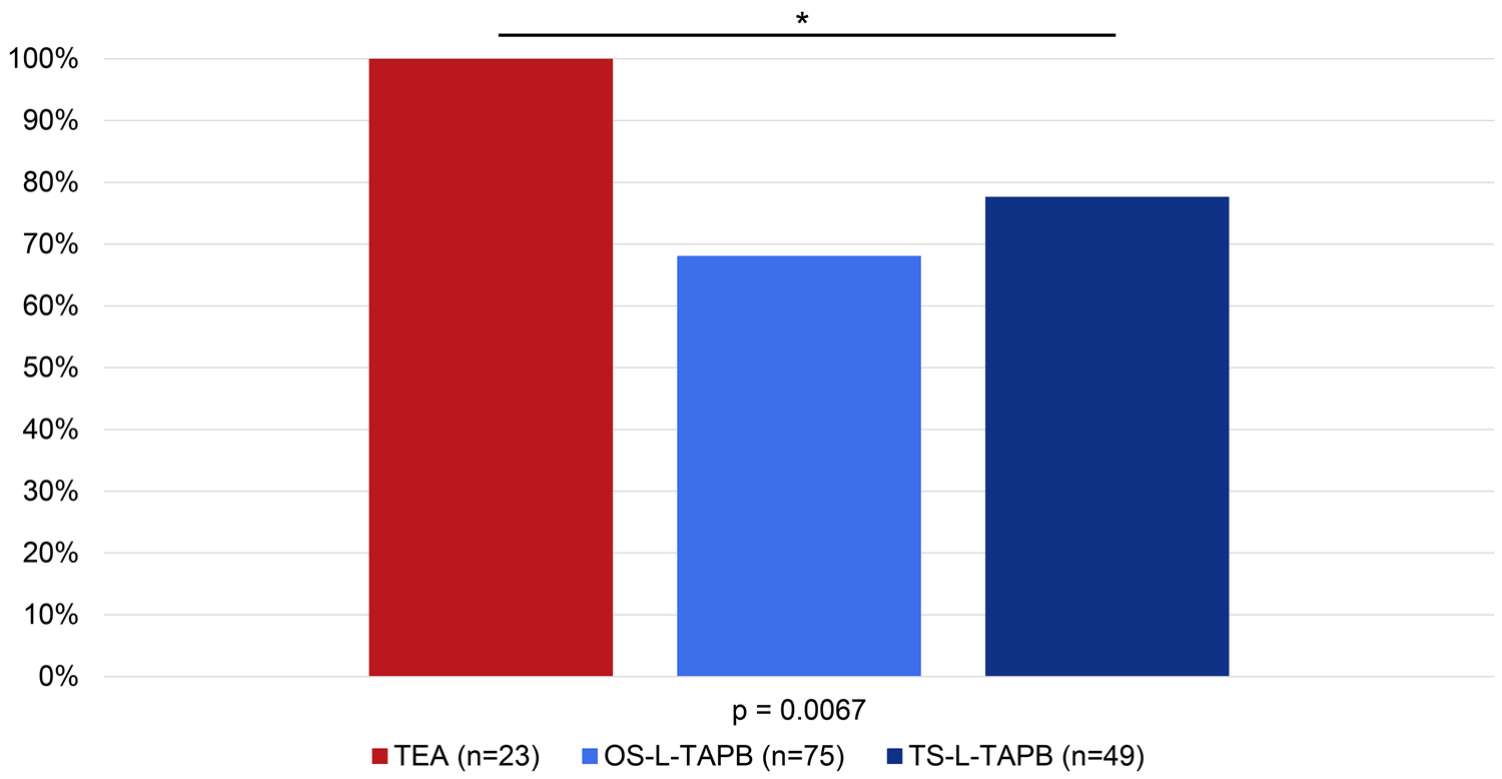
Oral and i.v. opioid-free patients in percentages in the recovery room within 1 h after arrival in the recovery room

One hour after arrival in the recovery room, significantly more patients in the TEA group (100%) did not need oral or i.v. opioids than in the TS-L-TAPB group (78%) and the OS-L-TAPB group (68%) (*p* = 0.0067) (Fig. 1).

The median MME for POD1-POD3 was always 0 mg in the TS-L-TAPB group, in contrast to the TEA and OS-L-TAPB groups. On POD1, the median MME showed no clinically or statistically significant differences: the OS-L-TAPB group had a higher MME with 2.8125 mg [0; 116.875] than the TS-L-TAPB group with 0 mg [0; 50] and the TEA group with 0 mg [0; 40] (*p* = 0.1774). On POD2, the median MME showed no clinically or statistically significant differences between all groups. However, the median MME tended to be higher in the TEA group with 5.625 mg [0; 85.625] than in the TS-L-TAPB group with 0 mg [0; 70] and the OS-L-TAPB group with 0 mg [0; 80] (*p* = 0.0675). On POD3, patients in the TEA group had a higher median MME with 40 mg [0; 80] than the TS-L-TAPB group with 0 mg [0; 80] and the OS-L-TAPB group with 0 mg [0; 80] (*p* = 0.0009).

**Fig. 2:**
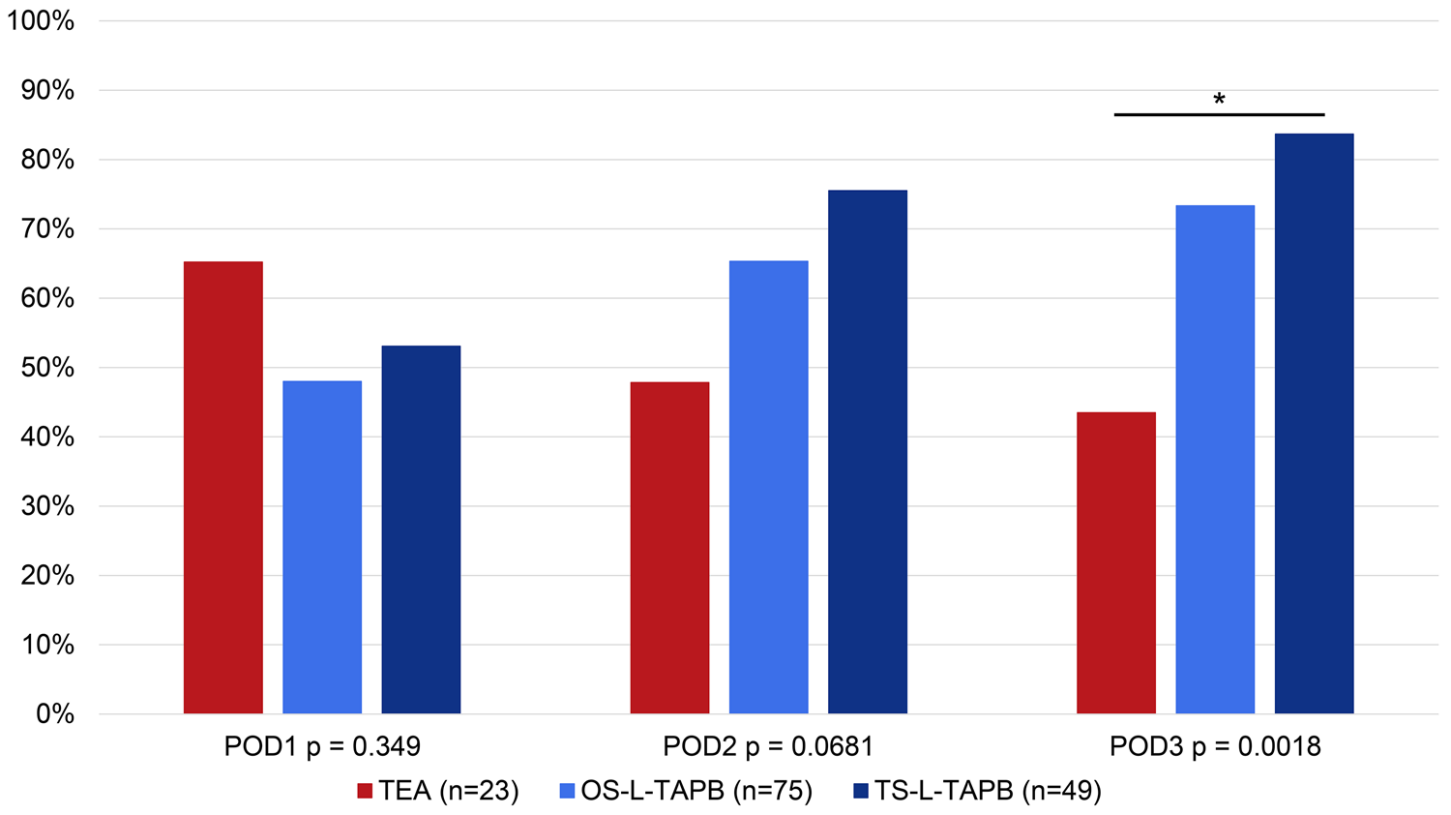
Oral and i.v. opioid-free patients in percentages per day

While the number of patients who needed oral or i.v. opioids increased daily in the TEA group (65%, 48%, 43%), more and more patients were no longer in need of oral or i.v. opioids in the OS-L-TAPB (48%, 65%, 73%) and TS-L-TAPB groups (53%, 76%, 84%). On POD3, significantly more patients in the TS-L-TAPB group (84%) were free of oral and i.v. opioid than in the OS-L-TAPB (73%) and TEA groups (43%) (*p* = 0.0018) (Fig. 2).

By the evening of POD1, the median MME requirement was statistically significantly lower in the TEA group with 0 mg [0; 40] than in the TS-L-TAPB group with 4.5 mg [0; 65.625] and the OS-L-TAPB group with 10 mg [0; 156.875] (*p* = 0.0037).

The median MME was significantly lower in the TEA group than in the OS-L-TAPB group. This clinically and statistically significant effect was lost by the evening of POD2, then both the TS-L-TAPB and the TEA groups had a median MME of 5.625 mg [0; 135.635, 0; 122.5] while the OS-L-TAPB group remained at 10 mg [0; 196.875] (*p* = 0.3588). In the evening of POD3, patients in the TEA group had a markedly higher MME with a median of 45.625 mg [0; 202.5] than patients in the OS-L-TAPB group with 10 mg [0; 245.625] and the TS-L-TAPB group with 5.625 mg [0; 215.625] but without reaching statistical significance (*p* = 0.1438).

Overall, 43% of patients from the TS-L-TAPB group did not require oral or i.v. morphine medication for up to 3 days postoperatively. This percentage however was not significantly higher than in the TEA (30%) and OS-L-TAPB groups (33%) (*p* = 0.4631). No postoperative cardiac problems such as arrhythmias occurred in the entire patient cohort.

The TEA group showed the longest LoS with a median of 7 days [5; 49]. This was significantly longer than for the TS-L-TAPB and the OS-L-TAPB group, where the median LoS was 5 days [3; 35, 3; 43] (*p* = 0.0018). There were no differences between groups regarding the need for metamizole and paracetamol in the first hour after arrival in the recovery room. The median requirement was 0 mg in all groups (*p* = 0.3853 and *p* = 0.8265).

## Discussion

The TAPB block has been used in clinical practice for more than 20 years since it was first described. Nevertheless, it has not yet become the standard in colorectal surgery. The ERAS-society initially favoured the TEA, but in the effort to make the patient independent as quickly as possible, the TAPB is gaining more and more use in elective bowel surgery.

Given the significant reduction in oral and i.v. opioid requirements over the first 3 postoperative days with the two-stage-laparoscopic TAP block compared with TEA, the results of our study support the hypothesis that two-stage L-TAPB (group TS-L-TAPB) may be an alternative to TEA in minimal-invasive elective bowel surgery. Furthermore, our results suggest that a correctly performed two-stage L-TAPB enabled the lowest cumulative oral and i.v. opioid consumption during the study period. In addition, the two-stage L-TAPB may potentially contribute to a further reduction in postoperative length of stay.

The oral and i.v. opioids requirements were lower in the TEA group on the day of surgery and on POD1; thereafter, they shifted in favour of the L-TAPB. This has also been shown in other studies. For example, Felling et al. showed that the patients with a TAPB had more pain than the TEA cohort until the evening of POD1. This trend reversed after this timepoint [19]. A similar trend was reported by Bumblyte et al. for the postoperative opioid requirement which was lower for TEA on the day of surgery and shifted in favour of TAPB from POD1 onwards [20]. The development of oral and i.v. opioid requirements in our study from POD1 onwards can be easily explained by the fact that in the recovery room and on POD1, the TEA flow, and thus the amount of opioids and local anaesthetics administered was adjusted to the patient’s pain sensation. Such an adjustment is not possible when a L-TAPB was performed. On POD2, the TEA-flow rate was generally reduced to achieve earlier removal of the catheter, which also explains the increased pain. Because of the removal of the TEA on POD 3 and the resulting increase in pain, the analgetic regimen was adjusted, such that a patient on POD 3 received a median of 40 mg morphine equivalent. The standard dose was 10 mg of oxycodone twice a day. It should be noted that because the TEA group was recorded retrospectively, we were unable to record TEA flow rates, and thus the additional opioid dose administered within the duration of TEA. Therefore, by the evening of POD2, patients in the TEA group had received far more opioids than the MME of oral and i.v. opioids that were recorded. Felling et al. showed that almost all patients with a TEA obtain most of their opioids via TEA [19].

In addition, compared with TEA, TAPB showed a reduction in hypotension [21], PONV [22], ileus [22], paraesthesia [22], urinary retention [23] and LoS [21, 23–25] in selected studies. Patients with a two-stage L-TAPB required fewer opioids during POD0-POD3 than patients with a one-stage L-TAPB. This suggests that two-stage L-TAPB may be superior to the one-stage L-TAPB, although no significant difference was found. Overall, this supports our assumption that the two-stage L-TAPB may be superior to the one-stage L-TAPB, even though no statistical significance was shown here. The advantages of a two-staged L-TAPB can therefore be achieved despite splitting the dose of the local anaesthetic. We assume that the first dose of L-TAPB ensures adequate analgesia for the patient during the surgery and in the recovery room. The second dose is supposed to reinforce and prolong this effect as well as allow the patient the longest possible duration of action of the L-TAPB after the operation.

In our study, we found a reduction in LoS in the TS-L-TAPB and OS-L-TAPB groups compared with the TEA group, as has been shown in other studies [21, 23–25]. However, it is important to note that LoS is a multifactorial endpoint and both performance and selection bias may influence it. For example, our ERAS-pathway certification for elective bowel resection, which began in January 2020 and was successfully completed in October 2021, may have had a bigger influence on LoS than the introduction of L-TAPB as one element of this pathway. In addition, significantly more rectal resections and stoma creations were performed in the TEA group, explaining longer LoS compared with colon resections without stoma creations.

Because meta-analyses also indicate that the analgesia of TAPB and TEA may be equivalent, it is necessary to consider how to optimise the TABP technique [21, 25]. Further studies should aim to prolong the duration of action of bupivacaine by improving its half-life (usually 8–10 h) and to determine the optimal injection sites.

In our study protocol, we chose three injections per side to provide optimal analgesia of the entire abdominal wall, including the incisions in the upper abdomen as well as the Pfannenstiel extraction site. Previous TAPB studies have pointed out the problem that a single lateral injection results in inadequate analgesia of the upper abdomen [26, 27]. For this reason, our data suggest that more than one injection per side may be useful for colorectal procedures to provide optimal analgesia of the entire abdominal wall.

In extensive minimally invasive procedures such as laparoscopic rectum resections, the main effect of long-acting local anaesthetics such as bupivacaine and ropivacaine may have worn off by the end of surgery. The duration of the postoperative analgesic effect of the TAPB has generally been described as 24 h [5, 28–30].

Future studies should address the optimal timing of L-TAPB. A possible RCT could answer the question of whether a preoperative, postoperative or two-stage L-TAPB provides the best outcome in terms of cumulative opioid consumption. In our study, we did not find a significant difference between using ropivacaine or bupivacaine for the TAPB. The question of which local anaesthetic is best suited for a long-acting and safe L-TAPB remains unanswered [30]. Furthermore, it should be clarified which additional adjuvant agents added to TAPB could prolong effective analgesia without causing an accumulation of side effects [31, 32]. The TAPB technique detailed above has not yet reached the ideal effectiveness. Further improvements will be possible when the optimal laparoscopically guided injection sites and technique, as well as the optimal timing and drug mixture, are tested in randomised trials.

## Limitations

The study is limited by possible performance and selection biases because of the retrospective data collection of the TEA and ropivacaine cohorts. Further limitations exist due to the partially heterogeneous patient group characteristics as well as differences in the performed surgical procedures. Since different local anaesthetics were used for the TS-L-TAPB and the OS-L-TAPB, a comparison is only possible to a certain extent even though they belong to the same group of local anaesthetics. In addition, the ERAS-certified bowel protocol had not yet been implemented in our hospital at the time the patients of our retrospective cohorts underwent surgery. Due to a staff shortage in 2020–2022, COVID-period patients could not be followed by an ERAS nurse during the prospective study period. This fact severely limits the comparative value of the LoS. While only correctly performed L-TAPB were included in the study analysis of the prospective cohort, the correctness of the performance of the L-TAPB using ropivacaine could not be verified in the retrospective cohort.

## Conclusion

The study results support the hypothesis that two-stage L-TAPB may reduce cumulative postoperative opioid requirements during the first 3 postoperative days compared to TEA. Hence, this surgeon-guided, easy-to-use technique may play an important role for improving colorectal enhanced recovery protocol implementation.

## Data Availability

The data that support the findings of this study are available on reasonable request from the corresponding author (Florian Herrle).
