# Correction to: Two-stage laparoscopic transversus abdominis plane block as an equivalent alternative to thoracic epidural anaesthesia in bowel resection—an explorative cohort study

**DOI:** 10.1007/s00384-024-04597-9

**Published:** 2024-01-29

**Authors:** M. Kaufmann, V. Orth, T.-J. Dorwarth, J. Benrath, B. Gerber, D. Ghezel-Ahmadi, C. Reißfelder, F. Herrle

**Affiliations:** 1 Department of Surgery, Medical Faculty Mannheim, University Medical Centre Mannheim, Heidelberg University, Mannheim, Germany; 2 Department of Anesthesiology, Medical Faculty Mannheim, University Medical Centre Mannheim, Heidelberg University, Mannheim, Germany

**Correction to: International Journal of Colorectal Disease (2024) 39:18** 10.1007/s00384-023-04592-6

To emphasise the shared first authorship, the authors' contributions have been supplemented accordingly. In addition, the sponsors of the open access publication were acknowledged.

Contributions:

“M.K. and V.O. wrote the main manuscript and are equally contributing authors."

In addition, we would like to thank our institution for funding the publication in the author contributions with the following sentence: "For the publication fee we acknowledge financial support by Deutsche Forschungsgemeinschaft within the funding programme "Open Access Publikationskosten" as well as by Heidelberg University."

The original article has been corrected.

